# Estimation and inference for the mediation effect in a time-varying mediation model

**DOI:** 10.1186/s12874-022-01585-x

**Published:** 2022-04-18

**Authors:** Xizhen Cai, Donna L. Coffman, Megan E. Piper, Runze Li

**Affiliations:** 1 Department of Mathematics and Statistics, Williams College, Williamstown, MA USA; 2 Department of Epidemiology and Biostatistics, Temple University, Philadelphia, PA USA; 3 Center for Tobacco Research and Intervention, School of Medicine and Public Health, University of Wisconsin, Madison, WI. USA; 4 Department of Medicine, School of Medicine and Public Health, University of Wisconsin, Madison, WI. USA; 5 Department of Statistics, Pennsylvania State University, University Park, PA USA

**Keywords:** Ecological momentary assessment, Intensive longitudinal data, Local linear regression, Nonparametric regression, Varying coefficient model

## Abstract

**Background:**

Traditional mediation analysis typically examines the relations among an intervention, a time-invariant mediator, and a time-invariant outcome variable. Although there may be a total effect of the intervention on the outcome, there is a need to understand the process by which the intervention affects the outcome (i.e., the indirect effect through the mediator). This indirect effect is frequently assumed to be time-invariant. With improvements in data collection technology, it is possible to obtain repeated assessments over time resulting in intensive longitudinal data. This calls for an extension of traditional mediation analysis to incorporate time-varying variables as well as time-varying effects.

**Methods:**

We focus on estimation and inference for the time-varying mediation model, which allows mediation effects to vary as a function of time. We propose a two-step approach to estimate the time-varying mediation effect. Moreover, we use a simulation-based approach to derive the corresponding point-wise confidence band for the time-varying mediation effect.

**Results:**

Simulation studies show that the proposed procedures perform well when comparing the confidence band and the true underlying model. We further apply the proposed model and the statistical inference procedure to data collected from a smoking cessation study.

**Conclusions:**

We present a model for estimating time-varying mediation effects that allows both time-varying outcomes and mediators. Simulation-based inference is also proposed and implemented in a user-friendly R package.

**Supplementary Information:**

The online version contains supplementary material available at (10.1186/s12874-022-01585-x).

## Background

Developments in mobile and wearable device technology have enabled the collection of intensive longitudinal data, [1] such as ecological momentary assessment (EMA), [2, 3]. EMA is particularly useful in health behavior change studies, for example, smoking cessation studies (see e.g., [4]), in which data on variables such as craving, withdrawal symptoms, or stress, are measured in real-time, real-world contexts. As the collection of data using EMA has grown, so have methods for analyzing and making the most of the temporal density of measurements, such as the mixed-effects location scale model [5] and the time-varying effect model [6]. EMA data captures temporal changes and, therefore, allows the estimation of time-varying effects. That is, the effect of one variable on another can vary as a function of time.

Often, the variables that are collected during EMA are variables that are targets of a behavior change intervention and are also thought to affect the health outcomes of interest. In other words, they are mediators, variables that lie on the pathway between the intervention and the outcome. However, there have been very few proposed methods for assessing mediation using this type of intensively measured data. Extensions of time-varying (i.e., varying-coefficient) models to mediation analysis would allow the estimation of time-varying mediation effects. For example, a pharmacological intervention may have an effect on cessation fatigue, defined as tiredness of trying to quit smoking [7], via negative affect but this effect may diminish as the time since quitting increases. As another example, a smoking cessation intervention may have an effect on remaining smoke-free via self-efficacy and this effect may strengthen as the time since quitting increases. Understanding time-varying mediation effects in treatment response is critical, as it will allow for tailoring of interventions, particularly as individuals transition from initial behavioral change to behavioral maintenance. This paper aims to propose an approach to mediation in which data on both the mediator and outcome variables are collected using EMA. Thus, values of the variables change over time and the effects of one variable on another may also change over time. Specifically, we propose a two-step approach to estimate the time-varying mediation effect. We develop a simulation-based approach to derive the corresponding point-wise confidence band for making statistical inferences regarding the time-varying mediation effect.

The rest of this paper is organized as follows. In Methods section, we present relevant background material on varying-coefficient models and the proposed model for time-varying mediation, including estimation and bootstrap inference. In Simulation studies section, we present simulation studies to examine the performance of the bootstrap confidence intervals. In Application: the Wisconsin smokers’ health study 2 section, we apply the proposed methods to data from a smoking cessation intervention study. In Discussion section, we discuss limitations, future directions, and conclusions.

## Methods

Time-varying coefficient models [8] have been used to model time-varying effects of an independent variable on a dependent variable [6, 9, 10]. These are essentially varying-coefficient models [11] applied to intensive longitudinal data. For each individual, *i*, the independent variable and the outcome variable are measured at multiple time points {*t*_*ij*_,*j*=1,2,…,*T*_*i*_}. The data collected are

$${}\{t_{ij}, X_{i}(t_{ij}), Y_{i}(t_{ij})\}, \quad\text{for} \quad i=1,2,\ldots,n,\quad j=1,2,\ldots,T_{i}.$$

The model can be written as

$$Y_{i}(t_{ij})={\beta}_{0}(t_{ij})+X_{i}(t_{ij}){\beta}_{1}(t_{ij})+ \epsilon_{i}(t_{ij}),$$

where *β*_0_(*t*) and *β*_1_(*t*) are time-varying coefficient functions and are assumed to be smooth functions of time. If needed, indicator functions can also be introduced to model population-level jumps at specific given change points. The error term *ε*(*t*) is a zero-mean stochastic process with covariance function, *γ*(*s*,*t*), between time *s*>0 and *t*>0. Not only are the effects (i.e., coefficients) of the predictor variables time-varying, but the values of the variables themselves also change over time. This is distinct from the commonly used mixed effect model which assumes a given functional form of the outcome variable and usually does not allow the coefficients expressing the effects of the covariates to change with time in non-parametric way, although the value of the covariates themselves may change with time [6, 8, 12]. The values of the variables themselves are not necessarily smooth functions of time, especially in the case of variables that have discrete values, but it is generally assumed to be so with continuous variables, such as the mediators and the outcome variables in the setting we introduce later. There are essentially two estimation approaches for time-varying effect models: splines and local smoothing methods (for a summary see [13]). In this paper, we focus on local smoothing methods, which locally approximate coefficient functions by linear or polynomial functions [14].

Fan and Zhang [15] proposed a two-step procedure that uses kernel methods to estimate the time-varying coefficients and their corresponding standard errors. Both simulations and real data applications showed the efficiency of their method over other previous proposals. This two-step procedure is computationally simpler than simultaneous estimation using spline methods, especially with longitudinal data sets. It especially fits well in our ILD setting, as the quality of the estimate in the smoothing step benefits from frequent time observations. This two-step procedure provides an important foundation for our proposed estimation procedure for time-varying mediation effects which combines the traditional linear mediation model estimation procedure and local polynomial smoothing.

Although time-varying coefficient models are relatively common for examining the time-varying effect of an independent variable on a dependent variable, relatively little work has examined time-varying effects for mediation. Lindquist [16] first introduced functional (or time-varying) mediation effects in which the independent and dependent variables were measured at a single point in time but the mediator was measured intensively over time using fMRI. More recently, VanderWeele and Tchetgen [17] proposed a mediation g-formula, which allows time-varying treatments, time-varying mediators, and an end-of-study point outcome. They mention the possibility of time-varying effects, but did not directly address them. However, in our application to a smoking cessation study, the mediator and outcome are both measured repeatedly over time (i.e., time-varying mediator and time-varying outcome) and the independent variable is random assignment to the intervention (not a time-varying treatment). An approach proposed by Bind et al. [18] uses the mixed-effects model to capture the time-varying effect. However, this still imposes some parametric restrictions on the shape of the time-varying effect, which may not be flexible enough and hence result in model misspecification. Thus, none of these previous models and estimation approaches apply directly to our smoking cessation study.

**Fig. 1:**
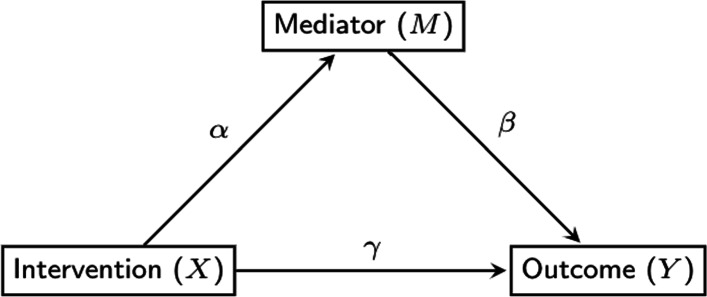
The traditional mediation model with time-invariant effects

Traditional methods of assessing mediation, shown in Fig. 1, generally specify the direct effect (i.e., the effect of the intervention on the outcome that does not go through the mediator) as *γ*, and the indirect or mediated effect as the product of paths *α* (i.e., the effect of the intervention on the mediator) and *β* (i.e., the effect of the mediator on the outcome)[19]. Note that this definition holds only for linear models in which the intervention does not interact with the mediator [20]. To test the statistical significance of the mediated effect, one can perform a Wald test using the asymptotic standard error formula introduced in [21], using the standard error calculated by a bootstrap procedure, or alternatively, constructing a confidence interval based on the percentiles of a non-parametric bootstrap distribution. Several prior simulation studies have shown bootstrap approaches to be superior, especially in smaller samples because $\hat {\alpha }\hat {\beta }$ may not be normally distributed [19, 22–25].

Mediation analysis has been extended to longitudinal data in which the mediator and/or outcome is measured repeatedly and therefore values on the variable itself may vary over time (see e.g.[19, 26, 27]); however, these extensions have not incorporated time-varying effects, which allow the direct and indirect effects to be summarized as functions of time rather than as a series or sum of single estimates at each measurement occasion. Such approaches work well for a few repeated measurement occasions but are cumbersome for intensive longitudinal data (ILD), such as that collected using mobile phones or other such devices that have allowed researchers to obtain more temporally dense data. For example, our empirical data analysis example from a smoking cessation study examines whether the intervention has an effect on cessation fatigue that is mediated by negative affect. Participants received morning and evening EMA prompts everyday to assess smoking, negative affect, withdrawal symptoms, and cravings over the course of 5 weeks. This measurement provides temporally dense ILD, such that mediation effects that vary as a function of time, rather than a single (i.e., constant over time) estimate of the effect, can be specified allowing for more complex, dynamic, hypotheses. In other words, mediation models can be specified that allow the effects of an intervention to vary over time, including the direct effect and the indirect effect.

**Fig. 2:**
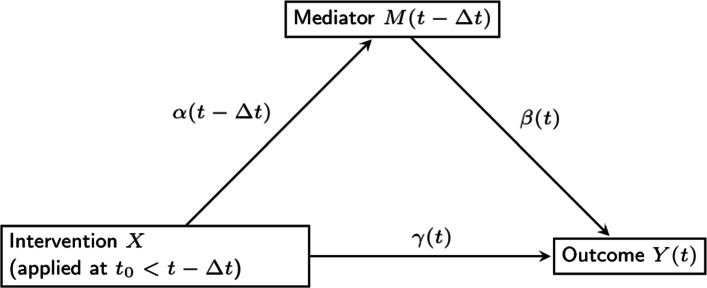
The proposed time-varying mediation model

Assuming linearity and no interactions between the intervention and mediator [20], as in traditional mediation analysis with time-invariant effects, the time-varying mediation effect can be defined as the product of two coefficients, but in this case, both coefficients are time-varying. That is, the two coefficients are no longer single numbers such as *α* and *β*; rather, they are functions of time, and the product term is also a function of time. Figure 1 is extended in Fig. 2 to include time-varying effects. In this paper, we propose to estimate the time-varying mediation model by extending the two-step approach [15], followed by bootstrapping to obtain confidence intervals for the indirect effect (i.e., the effect of the intervention on the outcome through the mediator).

### The proposed model

Extending the mediation framework in Fig. 1 to take advantage of the temporal density of ILD allows estimation of time-varying effects as shown in the dynamic mediation diagram in Fig. 2. In this model, we consider the measurement timing of the variables consistent with modeling mediation as a process that unfolds over time (i.e., intervention must precede change on the mediator, and mediator must precede change on the outcome). The intervention or independent variable, denoted *X*, is time-invariant, and assigned at time *t*_0_. Across time, the effect of *X* on the value of the mediator *M* at any time *t*>*t*_0_ (i.e., *M*(*t*)) is denoted by *α*(*t*). The value of the outcome variable *Y* at time *t* (i.e., *Y*(*t*)) is affected by the value of the mediator at a small window before time *t*, (i.e., *M*(*t*−*Δt*)). Here *Δt* is a small constant which represents the time-lag of the effect of the mediator on the outcome. More discussion of *Δt* will be presented shortly.

The diagram in Fig. 2 leads to the following time-varying mediation model:

$$\begin{array}{@{}rcl@{}} M(t)\! &=&\! \alpha_{0} (t)+I (t \geq t_{0}) \alpha (t) X+ \epsilon^{M}(t),\\ Y(t)\! &=&\! \beta_{0} (t)\,+\,I (t \geq t_{0}) \left\{\gamma(t) X \,+\, \beta(t) M(t-\Delta t) \right\}+ \epsilon^{Y}(t), \end{array} $$

where *ε*^*M*^(*t*) and *ε*^*Y*^(*t*) are both zero-mean independent stochastic processes. The time-varying mediation effect of interest is *α*(*t*−*Δt*)*β*(*t*). Because we do not impose any shape constraints on the smooth coefficient functions of the model, there are no restrictions on the functional form (e.g., linear, quadratic etc.) of the time-varying mediation effect. Suppose there are repeated measurements of *N* subjects at multiple time points {*t*_*ij*_}, then the observed data are

$${}\{X_{i}, (t_{ij}, M_{i}(t_{ij}), Y_{i}(t_{ij}))\}, \ i=1,2,\ldots, N, \ j=1,2,\ldots,T_{i},$$

and the model is

$$\begin{array}{@{}rcl@{}} M_{i}(t_{ij})\! &= &\! \alpha_{0} (t_{ij})+I (t_{ij} \geq t_{0}) \alpha (t_{ij}) X_{i}+ \epsilon^{M}_{i} (t_{ij})\\ Y_{i}(t_{ij})\! &=& \!\beta_{0} (t_{ij})\,+\,I (t_{ij} \geq t_{0}) \left\{\gamma (t_{ij}) X_{i} + \beta(t_{ij}) M_{i}(t_{ij} -\Delta t) \right\}\\ &&+ \epsilon^{Y}_{i} (t_{ij}). \end{array} $$

Note that all effects of the intervention, *X*_*i*_, are controlled by a post-intervention indicator, *I*(*t*_*ij*_≥*t*_0_), because the intervention is assigned at time *t*_0_. We presented these first set of equations that can be applied to a more general situation in which there may be repeated measurements before and after the intervention is assigned and the analyst may be interested in including both in the model. Since the mediation (i.e., indirect) effect is of primary interest, we focus on the time points after the intervention is assigned; thus, the indicator term can be dropped.

1 $$\begin{array}{*{20}l} M_{i}(t_{ij})\! &= \! \alpha_{0} (t_{ij})+ \alpha (t_{ij}) X_{i}+ \epsilon^{M}_{i} (t_{ij})  \end{array} $$

2 $$\begin{array}{*{20}l} Y_{i}(t_{ij})\! &= \!\beta_{0} (t_{ij})\,+\, \gamma (t_{ij}) X_{i} \!+ \!\beta (t_{ij}) M_{i}(t_{ij}\,-\,\Delta t)\,+\, \epsilon^{Y}_{i} (t_{ij}).  \end{array} $$

Recall that *Δt* is the time-lag in the mediator - outcome relationship. Ideally, its value, which reflects the true time difference in a mediation setting, should be determined by subject-matter knowledge, and preferably be reflected in the measurement timing in the design stage of the analysis [28]. In this case, we assume a small time difference, i.e. the value of the mediator *right before time t* predicts the value of the outcome variable at time *t*. Since this value is not observable, we use the value at the previous time point to approximate it during our estimations. This is a reasonable approximation in an ILD setting, where consecutive time points are close together. Additionally, models (1) and (2) are specified such that there are only two intervention groups (e.g., treatment versus control). That is, *X*_*i*_ is a binary indicator of the treatment condition, and the time-varying effect is the effect of the treatment as compared to the control group. For more than two intervention groups, the proposed model can be easily extended by adding more indicator variables (see the smoking cessation study in Application: the Wisconsin smokers’ health study 2 section as an example). Without loss of generality, we present the following proposed estimation procedure and bootstrap inference for the models in Eqs. (1) and (2).

### Estimation of the time-varying mediation effect

We could estimate the time-varying effect models in Eqs. (1) and (2) separately using the two-step estimation procedure [15] but here we propose a variant of that approach to estimate them simultaneously. Let {*t*_1_,*t*_2_,…,*t*_*T*_} be the distinct time points when the data are measured. For any fixed time point *t*_*j*_∈{*t*_2_,…,*t*_*T*_}, we observe complete data from *N*_*j*_ subjects (*N*_*j*_ does not necessarily equal *N*).Then for any individual *i* at this fixed time point *t*_*j*_, the observed data are

$$(X_{i}, M_{ij}, Y_{ij}), \quad i=1,2,\ldots, N_{j}, $$

where *M*_*ij*_=*M*_*i*_(*t*_*ij*_) and *Y*_*ij*_=*Y*_*i*_(*t*_*ij*_). Similar to the first step of the two-step procedure [15], at any fixed time *t*_*j*_, model Eqs. (1) and (2) are equivalent to the traditional linear mediation model at a single time point. Thus, we can estimate the value of the varying coefficient functions *α*(*t*_*j*_),*β*(*t*_*j*_), and *γ*(*t*_*j*_), which are treated as three parameters rather than three functions, by the least squares method, namely, by solving the following two optimization problems,

$$\begin{array}{*{20}l} &\min_{\alpha} \sum_{i=1}^{N_{j}} (M_{ij}-\alpha(t_{j}) X_{i})^{2}\ \text{and}\\ &\min_{\beta, \gamma} \sum_{i=1}^{N_{j}} (Y_{ij}-\gamma(t_{j}) X_{i} - \beta(t_{j}) M_{i,j-1})^{2}. \end{array} $$

Suppose the variables are mean centered or standardized at the fixed time point so that the intercept terms may be dropped. To derive a joint distribution of the estimated coefficients, we propose to combine the two least squares problems together to create a new least squares problem given as

3 $$ \begin{aligned} & \min_{\alpha, \beta, \gamma} \left\{\sum_{i=1}^{N_{j}} (M_{ij}-\alpha (t_{j}) X_{i})^{2} + \sum_{i=1}^{N_{j}} (Y_{ij}-\gamma (t_{j}) X_{i} - \beta (t_{j}) M_{i,j-1})^{2}\right\} \\ &\Leftrightarrow \min_{\boldsymbol{\delta}}\sum_{i=1}^{2N_{j}} (Y^{*}_{ij}-\boldsymbol{\delta}^{\top}(t_{j})\boldsymbol{X}^{*}_{ij})^{2} \end{aligned}  $$

where ***δ***(*t*_*j*_)=(*α*(*t*_*j*_),*γ*(*t*_*j*_),*β*(*t*_*j*_))^⊤^, and $Y^{*}_{ij}, X^{*}_{ij}$ in matrix forms are,

$$\begin{array}{@{}rcl@{}} \mathbf{Y}^{*}_{j} = \left(\begin{array}{c}M_{1j}\\M_{2j} \\ \vdots\\M_{N_{j},j}\\ Y_{1j} \\ Y_{2j} \\ \vdots \\ Y_{N_{j},j}\end{array}\right)_{2N_{j}\times 1} \mathbf{X}^{*}_{j} = \left(\begin{array}{ccc}X_{1} & 0 & 0 \\X_{2} & 0 & 0 \\ \vdots & \vdots& \vdots \\ X_{N_{j}} & 0 & 0 \\ 0 & X_{1} & M_{1,j-1} \\ 0 & X_{2} & M_{2,j-1} \\ \vdots & \vdots& \vdots \\ 0 & X_{N_{j}} & M_{N_{j},j-1} \end{array}\right)_{2N_{j}\times 3} \end{array} $$

Note that matrices $X_{j}^{*}$ and $Y_{j}^{*}$ depend on values of the mediators at both time *t*_*j*_ and time *t*_*j*−1_. In the case where not all *N*_*j*_ individuals observed at *t*_*j*_ also have values of the mediator observed at time *t*_*j*−1_, we will only use those with complete data to make the estimation at *t*_*j*_.

Denote the solution to the least squares problem in (3) as ***d***(*t*_*j*_)=(*a*(*t*_*j*_),*c*(*t*_*j*_),*b*(*t*_*j*_))^⊤^, that is, ***d*** is an estimate of ***δ*** and is a 3×(*T*−1) dimensional vector, which includes values of the estimated time-varying coefficient functions at all time points,

$$\begin{array}{*{20}l} {}\boldsymbol{d}&=\left(a(t_{2}),c(t_{2}),b(t_{2}), a(t_{3}),c(t_{3}),b(t_{3}), \cdots,\right.\\&\quad \left. a(t_{T}),c(t_{T}),b(t_{T})\right)^{\top} \end{array} $$

Similar to the second step of the two-step procedure [15], the coefficient functions $\hat {\alpha }$ and $\hat {\beta }$ in model Eqs. (1) and (2) are smoothed by local polynomial regression using (*a*(*t*_*j*_),*b*(*t*_*j*_)) *j*=2,3,...,*T* as,

4 $$\begin{array}{*{20}l} \hat{\alpha} (t- \Delta t) &= \sum_{l=2}^{T} w(t_{l}, t- \Delta t) a (t_{l}) \end{array} $$

5 $$\begin{array}{*{20}l} \hat{\beta} (t) &= \sum_{l=2}^{T} w(t_{l}, t) b (t_{l}) \end{array} $$

where *w*(*t*_*j*_,*t*) may be weights from any linear smoothing technique. Here, we use a local linear smoother, where the weight is defined as follows [15]

$$w(t_{j}, t)= \mathbf{e}^{\top}_{1, 2} (\mathbf{C}^{\top}\mathbf{WC})^{-1} {C}_{j}{W}_{j}, \quad j=2,3 \ldots T$$

where $\mathbf {e}^{\top }_{1, 2} = (1, 0), \mathbf {C}=(C_{2}, \ldots, C_{T})^{\top } \text { with } C_{j}=(1, t_{j}-t)^{\top }$ and $\mathbf {W}= diag(W_{2}, \ldots W_{T}) \text { with }W_{j}=K_{h}(t_{j}-t)=K(\frac {t_{j}-t}{h})/h$. The kernel function *K*(·,·) can be chosen to be any commonly used kernel (e.g., Gaussian) and the estimates are usually not sensitive to this choice [14]. The kernel function decides the importance of each neighborhood point (around the point to estimate) when fitting linear regressions locally. For example, the Gaussian kernel places more weight on the points which are closer to the point to estimate. In practice, researchers usually choose kernel functions with fast and easy computational implementations, such as the Guassian kernel and the Epanechnikov kernels [14]. The bandwidth *h* controls the scale of closeness to be used for these weights. A small bandwidth will produce a rough estimate which gives unnecessary bumps in the estimates due to individual data, while a bandwidth that is too large may over-smooth the data and hence miss important features or characteristics of the underlying curve. The bandwidth *h* can be chosen by an appropriate bandwidth selection method (e.g., rule of thumb) or other methods such as cross validation [14]. Then the desired mediation effect is

6 $$ \begin{aligned} \hat{\alpha} (t- \Delta t)\hat{\beta} (t) &= \left\{\sum_{l=2}^{T} w(t_{l}, t- \Delta t) a (t_{l}) \right\}\\ &\quad\left\{\sum_{l=2}^{T} w(t_{l}, t) b (t_{l})\right\}, \end{aligned}  $$

which can be rewritten as linear combinations of ***d***,

7 $$\begin{array}{@{}rcl@{}} \hat{\alpha} (t- \Delta t)\hat{\beta} (t) = (\mathbf{w}_{a}^{T}\mathbf{d})(\mathbf{w}_{b}^{T}\mathbf{d}),  \end{array} $$

where

$$\mathbf{w_{a}}= \left(\begin{array}{c} w(t_{2}, t- \Delta t)\\ 0\\ 0\\ w(t_{3}, t- \Delta t)\\ 0\\ 0\\ \vdots\\ w(t_{T}, t- \Delta t)\\ 0\\ 0\\ \end{array}\right), \text{and} \mathbf{w_{b}}=\left(\begin{array}{c} 0\\ 0\\ w(t_{2}, t)\\ 0\\ 0\\ w(t_{3}, t)\\ \vdots\\ 0\\ 0\\ w(t_{T}, t)\\ \end{array}\right).$$

### Estimating a bootstrap point-wise confidence interval for the mediation effect

To identify a statistically significant mediation effect in the time-varying setting, we consider the following hypothesis:

$$\begin{array}{@{}rcl@{}} &&H_{0}:{\alpha} (t- \Delta t){\beta} (t) = 0\text{ for any fixed }t\\ &vs.& H_{A}: \text{the mediation effect is not zero}. \end{array} $$

Since the distribution of the mediation effect is not necessarily normal, we use a nonparametric percentile bootstrap approach to construct confidence intervals at any given *t*. Specifically, we sample individuals from the data with replacement, and estimate the mediation effect at *t* using our proposed estimation procedure for each bootstrap sample. The lower and upper bounds of the 1−*α*% confidence interval are taken to be the corresponding lower (*α*/2) and upper (1−*α*/2) percentiles of the distribution of the estimated mediation effect from a large number of bootstrapped samples [29].

For any fixed time *t*, the above bootstrap percentile method creates a point-wise confidence interval for the mediation effect at that *t*. Connecting all confidence intervals yields a confidence band, but note that this is different from a simultaneous confidence band throughout the entire time interval, since the nominal confidence level is only satisfied at each fixed time point *t*. We return to this point in Discussion section. The estimation procedure and bootstrapped confidence intervals are implemented in an R package, *tvmediation*, that is available on CRAN.

## Results

### Simulation studies

To examine the performance of the proposed point-wise confidence interval, we consider the following two simulation models,

i. *α*_1_(*t*)=10+12*t*^3^,*γ*(*t*)=−20−18*t*,*β*(*t*)=50+150*t*^2^,
  *γ*(*s*,*t*)=15 exp(−0.3|*s*−*t*|)
ii. *α*(*t*)=15+8.7 sin(0.5*πt*),*γ*(*t*)=4−17(*t*−1/2)^2^,*β*(*t*)=1+2*t*^2^+11.3(1−*t*)^3^,*γ*(*s*,*t*)=15 exp(−0.3|*s*−*t*|)

The first model includes polynomial functions of different orders, and the second model incorporates a sin function to increase the complexity of the mediation effect. The two models are similar to those in Fan and Zhang [15] and Senturk and Muller [30].

Without loss of generality, observation times are generated as 50 equally spaced time points between 0 and 1. To generate the simulated data, we first randomly assign intervention and control group, each with probability of 0.5. The error term is generated from a multivariate normal distribution with mean zero and covariance function, *γ*(*s*,*t*), between time *s* and time *t*. It is a decaying exponential stationary covariance function and assumes a decreasing correlation with time. Similar covariance functions were used in [31] and [15]. The value of the mediator and the outcome variables are generated according to Eqs. (1) and (2). In the second step of the estimation procedure, local linear regression is used. We used the Gaussian kernel and the bandwidth is chosen by the rule of thumb formula in section 4.2 of Fan and Gijbels [14], where

$$\hat{h}_{ROT}=C_{\nu, p}(K)\left[ \frac{\hat{\sigma}^{2}_{Q}}{\sum_{i=1}^{n} \{ \hat{m}_{Q}(X_{i})\}^{2}}\right]^{1/5}$$

where $\hat {m}_{Q}$ is a fourth-order global polynomial fit of the data to be smoothed, $\hat {\sigma }_{Q}^{2}$ is the standardized residual sum of squares from this fit, and the constant *C*_*ν*,*p*_(*K*)=0.776 for local linear regression with the Gaussian kernel.

We consider three sample size conditions of *N*=100,200, and 500. To verify the nominal level for 95% confidence intervals, we calculated the coverage rate of the proposed point-wise confidence interval at *t*=0.2,0.4,0.6, and 0.8. Table 1 summarizes the results based on 1000 simulation replications.

**Table 1:** Coverage rate for 95% confidence intervals

	Sample Size	Coverage			
		t=0.2	t=0.4	t=0.6	t=0.8
Model i	100	0.95	0.95	0.94	0.94
	200	0.95	0.94	0.95	0.95
	500	0.94	0.94	0.94	0.95
Model ii	100	0.95	0.95	0.95	0.95
	200	0.94	0.94	0.93	0.93
	500	0.94	0.95	0.95	0.94

**Fig. 3:**
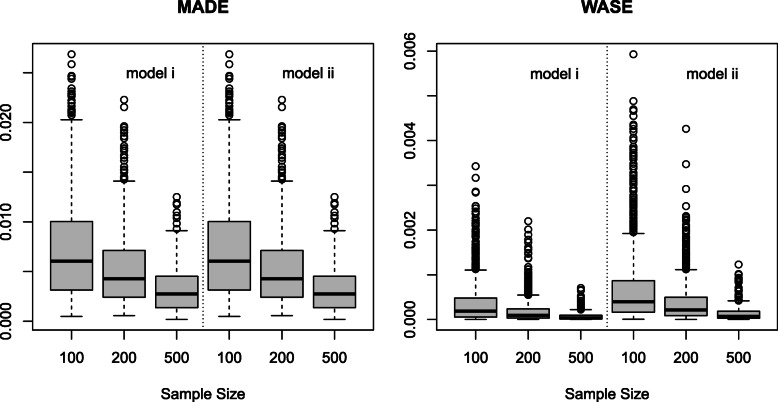
MADE and WASE for two models with different sample sizes. In each graph, the left three boxplots are for model i and the right three boxplots are for model ii

Except for a few settings, the coverage rates are all near a 95% confidence level at these points. We also evaluated the performance of the estimated time-varying mediation effect by the mean absolute deviation error (MADE) and weighted average squared error (WASE) [15, 30], defined as follows,

$$\begin{aligned} MADE &= (4T)^{-1} \sum_{j=1}^{T} \frac{|\eta(t_{j})-\hat{\eta}(t_{j})|}{\text{range}(\eta)}, \\ WASE &= (4T)^{-1} \sum_{j=1}^{T} \frac{\{\eta(t_{j})-\hat{\eta}(t_{j})\}^{2}}{\text{range}^{2}(\eta)} \end{aligned} $$

where *η*(*t*)=*α*(*t*−*Δt*)*β*(*t*) is the time-varying mediation effect. Figure 3 presents boxplots of these measurements for the two models. Not surprisingly, both of them show similar patterns with different sample sizes and models. Specifically, both MADE and WASE decrease as sample size increases for a particular model, and the error for model ii is slightly higher than that of model i.

**Fig. 4:**
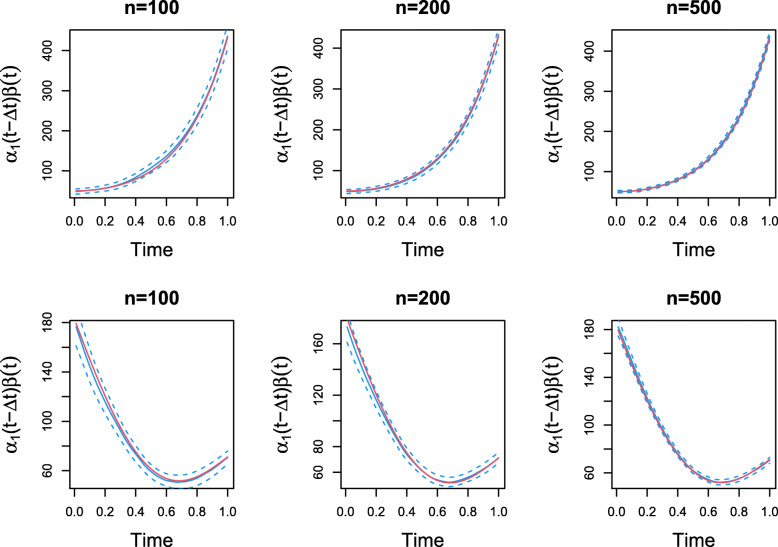
Point-wise confidence band of the time-varying mediation effect for the two simulation models. Plots in top row are for simulation model i, where the sample sizes are 100,200, and 500 from left to right. Plots in the bottom row for simulation model ii, where the sample sizes are 100,200, and 500 from left to right

To present a typical fit of the proposed procedure, we selected the simulation sample with MADE closest to the median value among all 1000 replications. The estimated time-varying mediation effect and the corresponding confidence intervals are plotted in Fig. 4, as compared to the true effect.

The red solid line is the true time-varying mediation effect, and the blue solid line is the estimated effect. For both models, the estimated effect is close to the true underlying effect. The blue dashed lines are the limits of the point-wise confidence band estimated by the proposed method. For both models, the width of the confidence band is not constant throughout the whole time range, but at each time, the true effect is fully contained in the confidence band. As the sample size increases, the confidence band becomes narrower. More simulation results to evaluate the coverage rate at additional new and observed time points, under different nominal confidence levels, and using under- or over- smoothed bandwidth selections are presented in the Supplementary material file. All results show that our method is performing well under different simulation settings.

### Application: the Wisconsin smokers’ health study 2

**Fig. 5:**
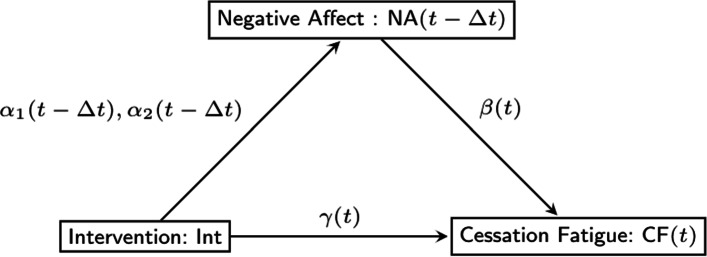
The time-varying mediation effect model for the smoking cessation study

We applied the proposed method to conduct an empirical analysis of data collected from a smoking cessation study, the Wisconsin Smoker’s Health Study 2, [32] which used EMA to assess negative affect and cessation fatigue during a smoking cessation trial. The study was a randomized comparative efficacy trial [32] directly comparing the two most effective smoking cessation therapies (varenicline and combination nicotine replacement therapy, which included use of nicotine patches and nicotine mini-lozenges) with one another and with an active comparator treatment (nicotine patch only). This dataset was also studied in [33] but with a different research question and different model for estimation. In total, 1086 smokers recruited from Madison and Milwaukee, WI were randomly assigned to one of the three 12-week pharmacotherapies. Participants completed one morning EMA prompt, and one evening prompt every day for one week prior to the quit day and for two weeks after the quit day and then every other day for the remaining two weeks of the EMA period (i.e., total of one week pre-quit and four weeks post-quit). Thus, there are 14 EMAs prior to the quit day and 42 after the quit day. The goal of our empirical analysis is to examine whether the intervention has an effect on cessation fatigue that is mediated by negative affect (see Fig. 5).

Cessation fatigue, defined as tiredness of trying to quit smoking [7], and negative affect, measured by asking participants if they were in a negative mood in the last 15 minutes, were both measured on 7-point Likert scales. Previous studies have found that negative affect and cessation fatigue are positively related and related to cessation failure [4].

We use data from the 42 EMAs post-quit day. Both the time-varying outcome, cessation fatigue (denoted CF_*ij*_), and time-varying mediator, negative affect (denoted NA_*ij*_), are assessed at each EMA prompt. Unlike in the simulation studies, and as is common in most empirical studies, especially with wearable and mobile devices, there are intermittent missing values in the data. Excluding individuals with no data at all, we have 1047 individuals in total, and the observed data are

$$\begin{array}{*{20}l} \{\text{Varen}_{i}, \text{cNRT}_{i},&\, (t_{ij}, \text{NA}_{ij}, \text{CF}_{ij})\},\quad i=1,2,\ldots,1047, \\ &j=1,2,\ldots,42. \end{array} $$

There are two indicator variables for the intervention: Varen _*i*_ indicates assignment to the varenicline group and cNRT _*i*_ indicates assignment to the combination nicotine replacement therapy group. The nicotine patch only condition is the reference group as it is considered the standard of care. Additionally, the observation times are not equally spaced (i.e., everyday for the first two weeks and every other day for the remaining two weeks). The previous proposed model can be modified to incorporate the additional intervention condition as follows:

8 $$\begin{array}{*{20}l} NA_{i}(t_{ij}) &= \alpha_{0} (t_{ij})+ \alpha_{1}(t_{ij}) Varen_{i}+ \alpha_{2}(t_{ij}) cNRT_{i}\\ &\quad+ \epsilon^{NA }_{i} (t_{ij})  \end{array} $$

9 $$\begin{array}{*{20}l} CF_{i}(t_{ij}) &= \beta_{0} (t_{ij})+ \gamma_{1} (t_{ij}) Varen_{i} + \gamma_{2}(t_{ij}) cNRT_{i}\\ &\quad+\beta(t_{ij}) NA_{i}(t_{ij}-\Delta t)+ \epsilon^{CF}_{i} (t_{ij})  \end{array} $$

**Fig. 6:**
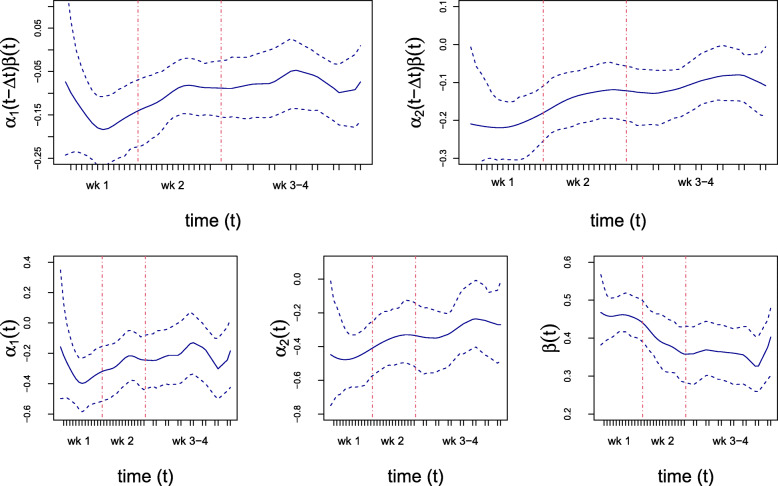
Time-varying mediation effects and individual effects. The top two plots display the mediation effects with the corresponding point-wise confidence intervals. Left panel is for the treatment varenicline, and the right panel is for the treatment combination nicotine replacement therapy, both as compared to the treatment of nicotine patch alone. The red vertical lines are separation of weeks. The three plots on the bottom row display the individual time-varying effects in the mediation model 8 and 9

Using the proposed method, the estimated mediation effects, *α*_1_(*t*−*Δt*)*β*(*t*) and *α*_2_(*t*−*Δt*)*β*(*t*), and the corresponding confidence bands are presented in Fig. 6. As compared to the nicotine patch only, the effect of varenicline on cessation fatigue that is mediated by negative affect becomes more negative shortly after quitting. More specifically, the *magnitude* of this negative mediation effect increases quickly in the first week after quit day, has a slower decrease in the second week, becomes stable in week 3, and slightly increases in week 4. The pattern for the effect of cNRT (vs. the nicotine patch only) was similar although the initial increase in the magnitude was not as pronounced but the mediation effect was statistically significant throughout the four weeks post-quit. Examination of the two time-varying effects that make up the mediation effect are also informative. Examination of the bottom row of Fig. 6 shows that varenicline (vs. the nicotine patch only) has a negative effect that becomes stronger over the course of the first week. This effect then begins to diminish during the following three weeks. In contrast, the strong negative effect of cNRT (vs. the nicotine patch only) on negative affect is apparent at the beginning of week one but then, similar to the varenicline group, diminishes during the following three weeks. Examination of the time-varying effect of negative affect on cessation fatigue reveals that there is a strong positive relationship (i.e., more negative affect results in more cessation fatigue) initially during the first week that diminishes over the following three weeks.

Additionally, compared to using nicotine patch only, the effects of varenicline and cNRT on cessation fatigue, as mediated by negative affect, are not only negative, but also, time-varying for the four weeks post quit day. Both mediation effects have a narrow confidence band and thus, we can rule out a constant mediation effect over time because we would not be able to fit a flat line over time within the confidence interval.

## Discussion

We have described a model for assessing mediation in the context of ILD in which both the mediator and outcome variables are time-varying. This model allows for estimation of time-varying mediation effects. ILD often arise from the collection of EMA data but may also arise from the collection of data from mobile devices, such as wrist-worn or hip-worn accelerometers. The temporal density of these data allow for more nuanced research questions that cannot be addressed by, for example, averaging over the EMA data and/or assuming that the mediated effect does not vary as a function of time. By allowing mediated effects to vary as a function of time, research questions such as the timing of important mediation effects can be assessed. Thus, our approach may prove useful to other researchers who wish to conduct mediation analysis in the context of ILD.

The simulation study showed that the proposed bootstrap pointwise confidence intervals contained the true time-varying mediation effect and that the estimated time-varying mediation effect was close to the true time-varying mediation effect. We then applied our approach to examine the mediation effect of three smoking cessation treatments (i.e., varenicline, cNRT, and nicotine patch only) on cessation fatigue via negative affect. The results indicated that the mediated effect 1) did indeed vary as a function of time, 2) was statistically different from zero throughout the four weeks post-quit day, and 3) that the effect was strongest in the first week post-quit for the varenicline group (vs. nicotine patch only). That is, the varenicline group experienced decreased negative affect during the first week, leading to decreased cessation fatigue. Interestingly, the effect was also strongest in the first week for the patch only group and the effect was immediate whereas the varenicline effect improved over the first half of the first week. The mediated effect for both treatments, compared to the patch alone, appeared to dissipate over the course of the first four weeks of the quit attempt. This information may lead to modifications and/or adaptations of the intervention to, for example, implement a behavioral component to address negative affect, with a specific focus on reducing negative affect in the first week of the quit attempt. This information would not have been evident had we assumed that the mediation effect was invariant across the four week post-quit period.

There are several recent studies which also examine the mediation effect in longitudinal settings. However, our approach is distinct from them in the underlying goal of estimating time-varying effects, and/or the method of estimation. For example, [34] proposes a latent growth curve model to study the mediation process but does not allow estimation of time-varying effects. Goldsmith et al. [35] proposed autoregressive and simplex models to study the longitudinal mediation effect but again do not allow estimation of time-varying effects. In contrast, our proposed model assumes that the observations come from continuous functions of time, and the effects are modeled as a smooth function of time, instead of a time series. We also distinguish our approach from that in Zhao et al. [36] by emphasizing the order of the mediator and outcome (i.e., mediator precedes outcome) in a longitudinal setting and by using a computationally efficient two-step estimation approach.

However, there are several limitations of the current approach. First, the proposed method only constructs a point-wise confidence interval. For inference at a fixed time point, a point-wise confidence interval is useful. However, a simultaneous confidence band is needed to make inferences over the entire time span. Thus, an obvious future direction is developing and estimating a simultaneous confidence band. Second, although the current approach does not require observations from all participants at all time points [15], the algorithm will not work if $\text {rank}(\mathbf {X}^{*}_{j}) <3$ (or <*d* in the general case). In such cases, one can implement the four methods discussed in Fan and Zhang [15] (see their Remark 1), or simply drop the observations at that time point. Third, the two-step method works well in the ILD setting. If there are data available from a very limited number of distinct time points, or the times of observation do not align well among subjects in the study, the estimates from the two-step procedure may not be appropriate or accurate. Finally, our proposed model does not include a lagged dependency on past values of the response variable to adjust for (time-varying) confounding. This is because in a longitudinal study, different subjects may be observed at different times, and the values of the response variable at the previous time point may not be well-defined, or have a different meaning for different individuals.

Mediation is inherently about causal pathways - the intervention has an effect on the mediator, which in turn has an effect on the outcome. In our particular application, individuals were randomly assigned to the smoking cessation treatments; however, they are not randomly assigned to the mediator and therefore, there may be confounders of the mediator and the outcome. In addition, due to the intensive longitudinal nature of the study, we cannot rule out the possibility of time-varying confounding. Of particular concern is the possibility of time-varying confounders of the mediator and outcome that have themselves been affected by the smoking cessation treatments. For example, the smoking cessation intervention may affect whether an individual smokes on a given day, which in turn affects both negative affect and cessation fatigue at a later time resulting in time-varying confounding. To infer causality, we would need to assume that there are no time-invariant unmeasured confounders of the mediator and outcome, additivity (no interactions or non-linearities), and no time-varying confounders of the mediator and outcome that have been influenced by the intervention. In addition, we assume temporal ordering such that the intervention occurs before the mediator which occurs before the outcome. These are the standard assumptions needed for a linear structural equation model to estimate a “causal" effect [16]. These are strong assumptions which investigators should consider the plausibility of in any particular application. Recent work in the causal inference literature has relaxed the time-varying confounding assumption to varying degrees - for example, by assuming the mediator depends only on recent values of a time-varying confounder [18]. Nevertheless, in our particular case, causal inferences regarding the total effect and the effect of the intervention on negative affect is possible due to randomization of the smoking cessation interventions.

## Conclusion

In conclusion, we have presented a model for estimating time-varying mediation effects which builds on previous work [15–17] to allow a time-varying outcome as well as a time-varying mediator. We also presented a method for obtaining point-wise confidence intervals for the product of two time-varying coefficient functions (i.e., a time-varying mediation effect), evaluated its feasibility in a small simulation study, and applied the method to evaluate the time-varying mediation effects of three pharmacotherapy smoking cessation interventions. We have implemented the estimation and bootstrap procedure in a user-friendly R package, *tvmediation*, available on CRAN. Although we cannot share the actual data, the R package contains data simulated to mimic the real data along with tutorials on how to use the functions to fit the model described above. We believe that this approach will be useful to those collecting ILD using mobile devices or self-reported EMA and who wish to examine mediation effects.

## Supplementary Information

**Additional file 1** Supplementary Material.

## Data Availability

The estimation and bootstrap procedure is implemented in a user-friendly R package *tvmediation* which is available on CRAN. The R package also contains data simulated to mimic the real data used in this paper along with tutorials on how to use the functions to fit the model described above. The original real data analyzed in the study are not publicly available due to privacy/ethical restrictions. For information on how to request the data, contact Megan E. Piper (mep@ctri.wisc.edu).

## Abbreviations

EMA: ecological momentary assessment
ILD: intensive longitudinal data
MADE: mean absolute deviation error
WASE: weighted average squared error
CF: cessation fatigue
NA: negative affect
cNRT: combination nicotine replacement therapy
