# Supplementary material for: Estimation and inference for the mediation effect in a time-varying mediation model
Source: BMC Med Res Methodol. 2022 Apr 18;22:113. doi: 10.1186/s12874-022-01585-x (PMC9014585; doi:10.1186/s12874-022-01585-x)
Supplement: Supplementary file 1 — Additional file 1 Supplementary Material. [file 12874_2022_1585_MOESM1_ESM.pdf]

Supplementary Material

This is the supplementary materials for the paper "Estimation and Inference for the Mediation Effect in a Time-varying Mediation Model". We include more simulation results to validate the performance of the proposed model and algorithm.

The first part of the results displays the empirical coverage rate for 95% confidence intervals at more evaluation time points within the time-span of the study. The model settings are the same as those in the simulation studies. The sample size is 200 and results for other sample size settings are similar. Table S1 gives the results at 10 equally spaced time points in $[0, 1]$, and Table S2 gives the results for all time points (50 equally spaced between 0 and 1) where simulated data were generated.

Table S1: Coverage rate for 95% confidence intervals at different evaluation time points

	Coverage									
time	0.05	0.15	0.25	0.35	0.45	0.55	0.65	0.75	0.85	0.95
Model i	0.937	0.945	0.939	0.939	0.941	0.947	0.950	0.942	0.947	0.949
Model ii	0.942	0.946	0.938	0.943	0.937	0.942	0.930	0.934	0.934	0.940

Table S2: Coverage rate for 95% confidence intervals at the same time points of observed data

	Coverage
time	0.010 0.030 0.050 0.071 0.091 0.111 0.131 0.151 0.172 0.192 0.212 0.232 0.252 0.273 0.293 0.313 0.333 0.353 0.374 0.394 0.414 0.434 0.454 0.475 0.495 0.515 0.535 0.556 0.576 0.596 0.616 0.636 0.657 0.677 0.697 0.717 0.737 0.758 0.778 0.798 0.818 0.838 0.859 0.879 0.899 0.919 0.939 0.960 0.980 1.000
Model i	0.938 0.940 0.937 0.938 0.937 0.943 0.943 0.945 0.941 0.944 0.943 0.940 0.940 0.941 0.940 0.939 0.942 0.939 0.942 0.943 0.944 0.945 0.940 0.940 0.942 0.939 0.942 0.948 0.946 0.946 0.947 0.949 0.949 0.946 0.948 0.946 0.942 0.940 0.944 0.942 0.943 0.947 0.948 0.946 0.946 0.947 0.948 0.949 0.949 0.950
Model ii	0.937 0.941 0.942 0.939 0.941 0.941 0.943 0.946 0.943 0.939 0.941 0.938 0.937 0.936 0.936 0.939 0.937 0.941 0.942 0.942 0.939 0.939 0.940 0.939 0.939 0.942 0.940 0.941 0.936 0.932 0.931 0.933 0.930 0.932 0.931 0.931 0.931 0.933 0.932 0.928 0.932 0.936 0.937 0.937 0.938 0.935 0.938 0.937 0.938 0.939

The following Table S3 gives the results for different nominal confidence levels, in additional to 95%.

Table S3: Coverage rate for confidence interval with different level of confidence

		Coverage			
	Level	t=0.2	t=0.4	t=0.6	t=0.8
	90%	0.89	0.89	0.90	0.90
Model i	95%	0.95	0.94	0.95	0.95
	99%	0.98	0.98	0.98	0.98
	90%	0.89	0.88	0.88	0.88
Model ii	95%	0.94	0.94	0.93	0.93
	99%	0.98	0.99	0.98	0.99

We performed two additional simulation studies, with the same settings as our original simulation studies, but now with a wider (3/2 original) or a narrower bandwidth (2/3 original). The comparisons are presented in the following Table S4, where h denotes the rule-of-thumb bandwidth selector used in the manuscript. The results shown in the table are for a sample size of 200; the results for other sample sizes were similar.

Table S4: Coverage rate for 95% confidence intervals with different bandwidths

		Coverage			
	Bandwidth	t=0.2	t=0.4	t=0.6	t=0.8
	(2/3)h	0.944	0.943	0.946	0.944
Model i	h	0.947	0.942	0.949	0.945
	(3/2)h	0.938	0.944	0.932	0.928
	(2/3)h	0.938	0.944	0.932	0.928
Model ii	h	0.943	0.938	0.929	0.930
	(3/2)h	0.940	0.941	0.933	0.929

Additionally, the following Figure shows the MADE and WASE: the accuracy evaluations for the mediation effect are very similar among the three choices of bandwidths.

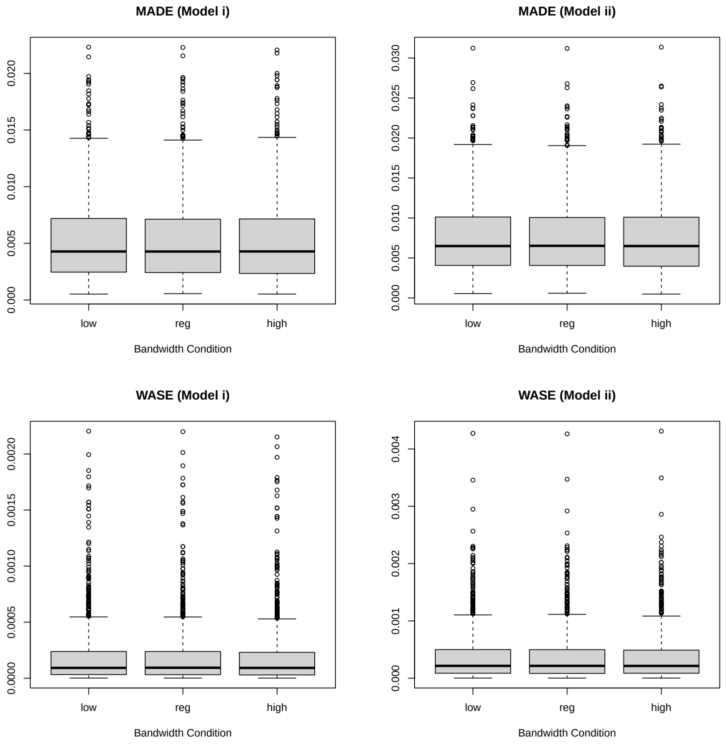

Figure S1: MADE and WASE results for comparing different bandwidth choices
